# A Novel Nonsense Mutation of the *GPR143* Gene Identified in a Chinese Pedigree with Ocular Albinism

**DOI:** 10.1371/journal.pone.0043177

**Published:** 2012-08-20

**Authors:** Naihong Yan, Xuan Liao, Su-ping Cai, Changjun Lan, Yun Wang, Xiaomin Zhou, Yan Yin, Wenhan Yu, Xuyang Liu

**Affiliations:** 1 State Key Laboratory of Biotherapy, Ophthalmic Laboratories, Department of Ophthalmology, Translational Neuroscience Center, West China Hospital, Sichuan University, Chengdu, People’s Republic of China; 2 Department of Ophthalmology, North Sichuan Medical College, Nanchong, People’s Republic of China; 3 Shenzhen Eye Hospital, Jinan University, Shenzhen, People’s Republic of China; Innsbruck Medical University, Austria

## Abstract

**Background:**

The purpose of this study was to elucidate the molecular basis of ocular albinism type I in a Chinese pedigree.

**Methodology/Principal Findings:**

Complete ophthalmologic examinations were performed on 4 patients, 7 carriers and 17 unaffected individuals in this five-generation family. All coding exons of four-point-one (4.1), ezrin, radixin, moesin (FERM) domain-containing 7 (*FRMD7*) and G protein-coupled receptor 143 (*GPR143*) genes were amplified by polymerase chain reaction (PCR), sequenced and compared with a reference database. Ocular albinism and nystagmus were found in all patients of this family. Macular hypoplasia was present in the patients including the proband. A novel nonsense hemizygous mutation c.807T>A in the *GPR143* gene was identified in four patients and the heterozygous mutation was found in seven asymptomatic individuals. This mutation is a substitution of tyrosine for adenine which leads to a premature stop codon at position 269 (p.Y269X) of GPR143.

**Conclusions/Significance:**

This is the first report that p.Y269X mutation of *GPR143* gene is responsible for the pathogenesis of familial ocular albinism. These results expand the mutation spectrum of *GPR143,* and demonstrate the clinical characteristics of ocular albinism type I in Chinese population.

## Introduction

Nystagmus is a common symptom of a range of diseases including at least three X-linked disorders, with one of those being ocular albinism type 1 (OA1; MIM 300500) mapped to Xp22.3 [1]. It should be distinguished from the congenital motor nystagmus (CMN), a hereditary disorder characterized by bilateral ocular oscillations that occurs in the absence of any obvious ocular disorders [2]. Identification of the underlying disease of CMN often requires extensive clinical, electrophysiological, psychophysical, and eventually molecular genetic examinations, especially when clinical findings are unrevealing [3], [4]. The prevalence of X-linked OA1 is estimated to be 1 in 50,000 live births. Most male patients with OA1 showed normal skin and hair pigment, but will usually have signs and symptoms of ocular albinism, including nystagmus, poor visual acuity, iris translucency, foveal hypoplasia and albinotic fundus [5], [6]. However, the characteristics of OA1 have not been well defined in Asians. OA1 is caused by mutations in the G protein-coupled receptor 143 (*GPR143*) gene, originally also known as the *OA1* gene [7]. Various types of mutations in GPR143 have been identified in different countries, but X-linked OA1 in the Chinese population was rarely reported [8].

In the present study, a five-generation Chinese family with OA1was recruited. All affected individuals exhibited nystagmus as the main symptom and failed to show photophobia, iris translucency and strabismus. This pedigree was initially considered as congenital nystagmus. Diagnosis of OA1was made by extensive clinical examinations. Four-point-one (4.1), ezrin, radixin, moesin (FERM) domain-containing 7 (*FRMD7* candidate gene for CMN) and G protein-coupled receptor 143 (*GPR143*, candidate gene for OA 1) genes were analyzed.

## Methods

### Family Recruitment

**Figure 1:**
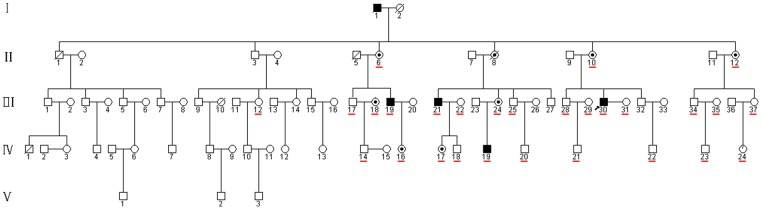
The pedigree of X-linked OA1. A filled square indicates an affected male and a dot in the middle of the circle indicates a carrier. The proband is marked by an arrow. The underline indicates family members enrolled in this study.

A five-generation Chinese family with OA1 was recruited in Sichuan (Figure 1). Written informed consent was obtained in accordance with the Declaration of Helsinki before blood samples were taken for analysis (see attachment for details). Three minors were used in this study. Written informed consent was obtained from the guardians on behalf of the minors (see attachment for details). This study was approved by both West China Hospital, Sichuan University Institute Review Board and North Sichuan Medical College Institute Review Board.

### Clinical Examination

Complete physical examination and detailed ophthalmological examination were carried out on the subjects of this family, including 4 affected patients, 7 carriers and some asymptomatic individuals. Visual acuity (VA) was measured using the best-corrected Snellen visual acuity test. Fundus and OCT examinations were performed using fundus camera (nonmyd WX 3D, Kowa, Japan) and Spectralis OCT (Heidelberg Engineering, Germay).

### Mutation Screening and Sequence Analysis

Genomic DNA was extracted from 200 µl of peripheral blood using the standard phenol/chloroform method. DNA integrity was evaluated by 1% agarose gel electrophoresis.

Mutation analysis of *FRMD7* and *GPR143* was performed using PCR and direct sequencing. Primers were designed to cover the sequences of all coding exons of the genes, according to published primer sequences with some modification (Table 1) [9], [10]. The primers were synthesized by Invitrogen (Carlsbad, CA, USA). PCRs were carried out in a MyCycler thermocycler (Bio-Rad, Hercules, CA, USA) using the following program: initial denaturation at 95°C for 2 min followed by 35 cycles of 94°C for 10s, 54°C–56°C for 30s, and 72° for 1 min, and then a final extension at 72°C for 5 min.

PCR products were directly sequenced using an ABI 377XL automated DNA sequencer (Applied Biosystems, Foster City, CA), and analyzed with the DNAStar (Madison, WI) software and compared with the published *FRMD7* and *GPR143* sequences. Mutation was named according to the nomenclature recommended by the Human Genomic Variation Society (HGVS).

**Table 1:** Primers used in PCR for amplification of *FRMD7* and *GPR143*.

Exon	Primer direction	Sequence (5′→3′)	Annealing temperature (°C)	Product size (bp)
1	FRMD7-1F	GAGAAAGCACCCACAGCACT	56	229
	FRMD7-1R	CAGTCCTCCTTCATTCAGTCC		
2	FRMD7-2F	TGTGGCTTCTACCCTTTATTC	54	403
	FRMD7-2R	AGTGTTGGGATTACAGGCAT		
3	FRMD7-3F	TGGAGCAGTGATTCAAATGTC	54	336
	FRMD7-3R	TCTAACTGTGAACTCTCTTCCT		
4	FRMD7-4F	CCTATAACTGTTGTGATGGAC	54	260
	FRMD7-4R	CATCTCCCAGACAGTGACTTA		
5	FRMD7-5F	TGCCAAAGTGTTCAATCAGC	54	368
	FRMD7-5R	CTCCTGTGCTTGGTTCTCTA		
6+7	FRMD7-6F	TGGAGGACAAGGGTATGCT	55	642
	FRMD7-6R	GTGATAATACTGAGGGGTGAG		
8	FRMD7-7F	GACCACAGCTCCTACCCAGT	56	374
	FRMD7-7R	AAAGACACACCATCACTCAGG		
9	FRMD7-8F	GAGCAATAGTTTGGAAGGCAT	54	293
	FRMD7-8R	AAGAAGCAGTGTGAGCAGTTT		
10+11	FRMD7-9F	TGTTCTCTGCCTGGTCCTTG	55	532
	FRMD7-9R	TTTACACACTGGGATTCTGG		
12	FRMD7-10F	CTACCCTAGAATAGAACATGGA	54	702
	FRMD7-10R	ATTCCTTGGGCTTCTTTCAG		
12	FRMD7-11F	GGAAAGGACAAGTCCACATA	54	660
	FRMD7-11R	TTCTGCCTAAGTCGGTAACA		
1	GPR-1F	AACCTTCCCAACCTTTCTGC	55	698
	GPR-1R	CCTCTCGTCCTCACTCCATC		
2	GPR-2F	CAGTGAGCAGGGTTTTTACCA	55	537
	GPR-2R	AACAGACTCCCAGGGTTTGC		
3	GPR-3F	GTCTACCCTGCCGTCTCAAG	56	334
	GPR-3R	TGAGCTGCTGTGGATGTTTC		
4	GPR-4F	CTCAGCAGCACGAGGAAACT	56	465
	GPR-4R	ACAAACGAGAAAGGCAGAGC		
5	GPR-5F	CTTAGGGGTCCTCCCATTTC	55	575
	GPR-5R	TGGCACTGAGCTAACAAACG		
6	GPR-6F	TCAGTGACTTGCTTTGCTTCCT	54	387
	GPR-6R	TCCTCAAAGGGCACCTAGCA		
7	GPR-7F	GCACCTGGCCCTCTTAGTTT	55	458
	GPR-7R	ACCTGTAGTCCCAGTTACTCA		
8	GPR-8F	ATGGTCCCTTCCAAGCGAGT	56	494
	GPR-8R	GTTCACATGAGAGGTGCTGCT		
9	GPR-9F	ACTCCATGCACTGAATACTGA	54	485
	GPR-9R	GGATGTGGACCTTACACTTACT		

## Results

### Clinical Phenotype

**Figure 2:**
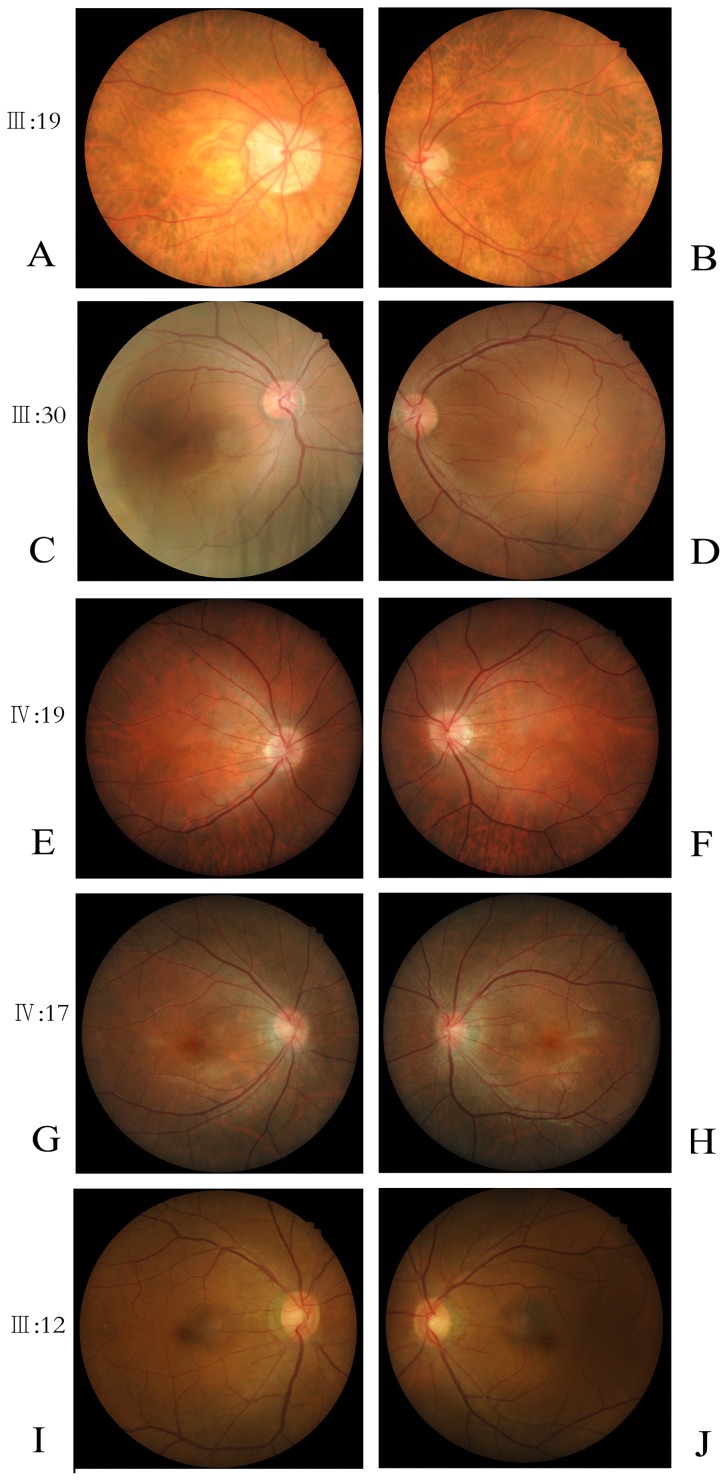
Fundus photographs of three patients, one carrier and one normal individual. A–F: three patients; G–H: one carrier; I–J: one normal individual.

**Figure 3:**
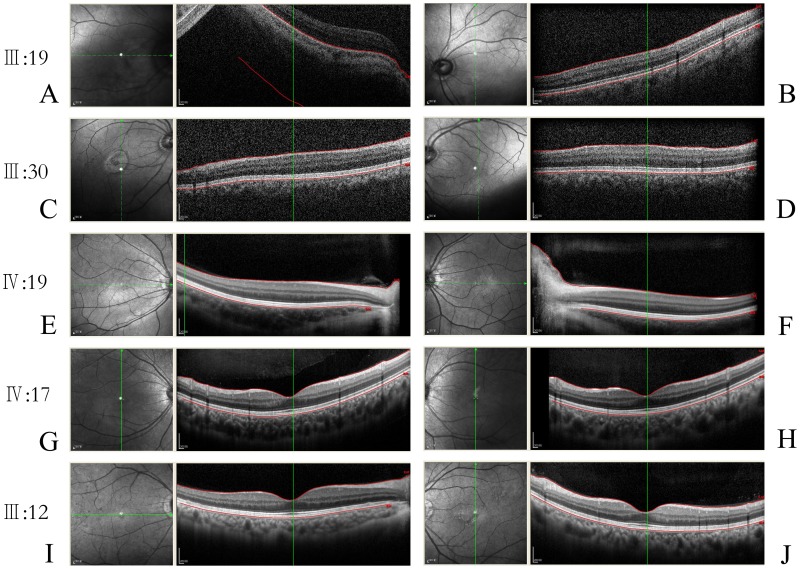
OCT test photographs of three patients, one carrier and one normal individual. A–F: three patients; G–H: one carrier; I–J: one normal individual. It should be noted that detailed structural imaging of the fovea was not successfully obtained in two patients (III:19 and III:30) with more severe nystagmus due to their poor fixation. Foveal image of one patient (IV:19) with relatively mild nystagmus was obtained.

In this five-generation Chinese family, the disease was transmitted from female carrier to affected son, indicating that the disease was inherited in an X-linked recessive pattern (Figure 1). The clinical characteristics of OA1 in this pedigree are described as in Table 2, Figure 2 and 3.

### Reduced Visual Acuity and Nystagmus

All four patients presented with nystagmus and reduced visual acuity (corrected visual acuity of 0.01–0.2). The nystagmus was present during their first three months after birth. Eye movement recording revealed that the patients had conjugate horizontal nystagmus. The proband (patient III:30, Figure 1), a forty-two-year-old male, presented with nystagmus with best corrected visual acuity being 0.2 OD and 0.2 OS. He presented with nystagmus and congenital cataract on the fortieth day after birth and underwent cataract extraction and intraocular lens implantation at the age of 41. No pigmentation abnormality of skin and hair was observed in the participants. Ocular abnormalities were not found in other asymptomatic members examined in this family.

### Presence of Hypopigmentation in the Fundus and Foveal Hypoplasia

Compared with normal individuals (Figure 2, I, J), the patients (III:19, IV:19) exhibited an albinotic fundus (Figure 2, A, B, C, D, E, F). All of the patients had foveal hypoplasia. The OCT showed extension of all neurosensory retinal layers through the area in which the fovea would normally be located (Figure 3). The clinical features of affected males and female carriers were shown in Table 2.

**Table 2:** Summary of clinical features of affected males and female carriers.

ID	Gender	Age	Visual acuity (left/right)	Iris hypopigmentation	Fundus hypopigmentation	Fundus foveal hypoplasia	Nystagmus	Mutation
patients								
III:19	Male	47	0.05/0.1	No	Yes	Yes	Yes	hemizygous
III:21	Male	47	0.01/0.01	No	Yes	Yes	Yes	hemizygous
III:30	Male	42	0.2/0.2	Mild	No	Yes	Yes	hemizygous
IV:19	Male	21	0.1/0.1	No	Yes	Yes	Yes	hemizygous
Carriers								
II6	Female	78	0.05/0.05	No	Cataract, Unclear fundus	Cataract, Unclear fundus	No	heterozygous
II:10	Female	64	0.8/0.8	No	No	No	No	heterozygous
II:12	Female	62	0.6/0.6	No	No	No	No	heterozygous
III:18	Female	55	0.7/0.7	No	No	No	No	heterozygous
III:24	Female	44	0.8/0.8	No	No	No	No	heterozygous
IV:16	Female	20	1.0/1.0 (best corrected)	No	Yes (High myopia)	No	No	heterozygous
IV:17	Female	24	0.8/0.8	No	No	No	No	heterozygous

### *GPR143* Mutation Identification and Analysis

A novel nonsense mutation, c.807T>A, at codon 807 (TAT to TAA) of exon 7 in *GPR143* gene was identified in all affected males. This mutation was presented as heterozygous in all obligate female carriers, and it was not found in normal members of the family. The c.807T>A mutation caused a substitution of tyrosine leading to a premature termination codon at position 269 (p.Y269X) of GPR143 protein.

## Discussion

A Chinese family with “congenital” nystagmus as the main symptom was reported in this study. There is no difference in iris pigmentation between patients (except the proband) or carriers and normal individuals in this family. The patients presented with only mild hypopigmentation in fundus. The presence of foveal hypoplasia could be ignored since the macular morphology could not be easily obtained by OCT due to the poor fixation of the nystagmus eye of the patients. Therefore this Chinese family was considered originally as congenital nystagmus. Preising *et al.* reported that nystagmus, macular hypoplasia and hypopigmentation of the fundus were the characteristic signs of ocular albinism which are more reliable in identifying patients with albinism [6], [11].

For the molecular diagnosis of this pedigree, the *FRMD7* gene (candidate gene for congenital nystagmus) and *GPR143* gene (candidate gene for OA1) were analyzed. The sequence analysis of *GPR143* demonstrated a novel nonsense mutation (p.Y269X) in exon 7. All affected males were hemizygous for the mutation and female carriers were heterozygous for the mutation whereas the other normal members of the family had no mutation. Another gene, *FRMD7*, involved in the development of congenital nystagmus, was screened and no mutation was found. Thus, the results of clinical and genetic findings provide solid evidence showing that this Chinese family has X-linked OA1, and the p.Y269X mutation of *GPR143* is responsible for the pathogenesis.

Bassi *et al.* (1995) cloned *GPR143* gene for ocular albinism type 1 from the distal short arm of the X chromosome [1]. Also in 1995, Schiaffino *et al.* screened *GPR143* gene and detected various mutations in one-third of X-linked ocular albinism (XLOA) patients [7]. To date, about one hundred mutations of *GPR143* were deposited in Human Gene Mutation Database (HGMD), including deletion, frameshift, and nonsense mutations. Most of *GPR143* mutations were reported in a large collection of patients mainly with ocular albinism [12], [13], [14], [15]. In 2001, Preising *et al.* reported an X-linked CN family with ocular albinism and found 14 bp deletion in *GPR143* gene [6]. In 2007, Liu *et al.* identified a novel missense *GPR143* mutation in a large Chinese family with CN as the most prominent and consistent manifestation [16]. In more recent years, *GPR143* mutations have been identified in the other two Chinese families with X -linked CN without any classical phenotype of OA1. One family had a 37 bp deletion mutation in exon 1 of *GPR143* [17]. The other family had a 19 bp duplication in exon 1 of *GPR143* and all affected individuals exhibited nystagmus [10]. These two reports did not present sufficient clinical data to evaluate their hypothesis of isolated nystagmus from *GPR143* variants. Preising *et al.* suggested male patients with congenital nystagmus were candidates for X-linked OA and a thorough clinical examination was needed [11]. Furthermore, analysis of the *FRMD7* and *GPR143* genes would be helpful to distinguish these two conditions from the molecular level.

*GPR143* on chromosome Xp22.3 contains 9 exons and encodes a protein of 404 amino acids containing seven putative transmembrane domains and one potential N-glycosylation site using an asparagine at codon 106 [18]. *GPR143* protein is a conserved integral membrane protein that has weak similarities to G protein-coupled receptors (GPCRs), which participate in the most common signal transduction system at the plasma membrane. It binds heterotrimeric G proteins, which suggests that *GPR143* GPCR-mediated signal transduction systems also operate at the internal membranes in mammalian cells [19]. The p.Y269X mutation identified in our study was predicted to result in a truncated protein with 269 amino acids shorter than the normal full-length protein, suggesting that this is a loss-of-function mutation. Furthermore, the mutated transcript is likely to be degraded by the nonsense-mediated mRNA decay (NMD) pathway. This hypothesis assumed that the truncated protein may not even be produced.

It is unclear how the mutated *GPR143* causes the ocular abnormalities, such as macular hypoplasia in people with ocular albinism. Lopez *et al.* proposed that L-3, 4-dihydroxyphenylalanine (L-DOPA) might be a ligand for the protein encoded by *GPR143* [20]. L-DOPA is a precursor in melanin synthesis that has been considered as an antimitogenic factor in cell cycle regulation, playing a crucial role in the maturation of the retina and the optic nerve [21], [22]. *GPR143* is not involved in the production of melanin itself, but rather the pigment distribution or production of a precursor like L-DOPA as a cause of developmental anomalies of macular development. The impaired macular development might then cause the vision loss and nystagmus [11], as reported in this study.

The ocular disorders should be eliminated before the diagnosis of congenital motor nystagmus can be made. However, some diseases such as OA1 can be ignored or misdiagnosed. In general, OA1 is characterized by presence of photophobia, congenital nystagmus, strabismus, iris translucency, hypopigmentation of the ocular fundus, foveal hypoplasia, and impaired vision [23]. In African-American males, iris color is usually brown with little iris translucency (compared to Caucasians where iris translucency is more common) and individuals with darker skin may have scattered hypopigmented macules, but this is rarely seen in the skin of Caucasian individuals [24], [25]. The characteristics of OA1 have not been well defined in Asians. There were Chinese pedigrees of congenital nystagmus reported showing that the patients in these families had very similar symptoms as those in our pedigree. It is interesting to reevaluate these pedigree to make sure the diagnosis of “congenital nystagmus”, not “ocular albinism”, is correct, and the impression that OA1 has rarely seen in China is correct.

In summary, this study adds a novel nonsense mutation to the existing spectrum of *GPR143* mutations in Chinese families with X-linked OA1. The specific molecular mechanism by which these *GPR143* mutations result in OA1 is still unknown, further functional studies are needed to provide new insights to this inherited ocular disease.
